# Silencing of Kinesin Light Chain 1 Suppresses Aggressive Phenotypes in Cholangiocarcinoma Cells Through Transcriptomic Alterations

**DOI:** 10.3390/ijms27177525

**Published:** 2026-08-22

**Authors:** Thanakrit Rattanaarchanai, Phonprapavee Tantimetta, Phanthipha Runsaeng, Sompop Saeheng, Sumalee Obchoei

**Affiliations:** 1 Department of Biochemistry, Faculty of Science, Prince of Songkla University, Hat Yai 90110, Songkhla, Thailand; 2 Center of Excellence for Biochemistry, Faculty of Science, Prince of Songkla University, Hat Yai 90110, Songkhla, Thailand

**Keywords:** kinesin light chain 1, cholangiocarcinoma, RNA sequencing, tumor progression, MAPK signaling pathway

## Abstract

Cholangiocarcinoma (CCA) is an aggressive malignancy with limited treatment options and poor clinical outcomes. Kinesin light chain 1 (KLC1), a component of the kinesin-1 motor complex involved in intracellular transport, has been implicated in cancer biology; however, its role in CCA remains unclear. This study investigated the functional role and molecular alterations associated with KLC1 silencing in CCA. Analysis of publicly available datasets showed that KLC1 mRNA expression was significantly elevated in CCA tissues, and immunohistochemical images from the Human Protein Atlas demonstrated stronger KLC1 protein expression in tumor tissues. siRNA-mediated KLC1 knockdown markedly suppressed cell proliferation, migration, and invasion in KKU-213A and KKU-055 cells and altered the expression of epithelial–mesenchymal transition-associated proteins. Transcriptomic profiling identified 2074 differentially expressed genes following KLC1 knockdown. Functional enrichment analyses revealed significant alterations in cytoskeleton-associated processes and mitogen-activated protein kinase (MAPK) signaling. Protein–protein interaction network analysis identified interconnected gene networks associated with these pathways. Selected differentially expressed genes were validated by RT–qPCR, supporting the transcriptomic findings. Collectively, these results suggest that KLC1 contributes to aggressive phenotypes in CCA cells and is associated with transcriptomic alterations involving cytoskeletal regulation and MAPK signaling, highlighting KLC1 as a potential contributor to CCA progression and warranting further investigation.

## 1. Introduction

Cholangiocarcinoma (CCA) is a highly aggressive malignancy arising from the biliary epithelium and is characterized by marked clinical, histological, and molecular heterogeneity, contributing to its poor prognosis and limited therapeutic success [1,2]. The incidence and mortality of CCA have been increasing globally, particularly in endemic regions such as Southeast Asia, where risk factors including chronic inflammation and liver fluke infection play critical roles in tumorigenesis [2,3]. Despite advances in surgical techniques and systemic therapies, most patients are diagnosed at advanced stages when curative treatment is no longer feasible, and current therapeutic strategies provide only modest survival benefits [1,4]. At the molecular level, CCA development is driven by genetic and epigenetic alterations, including dysregulation of oncogenic pathways such as MAPK, PI3K/AKT, and Notch signaling, which promote tumor progression, metastasis, and therapeutic resistance [5]. Emerging evidence also highlights the role of the tumor microenvironment and epithelial–mesenchymal plasticity in facilitating cancer cell invasion, dissemination, and immune evasion [6]. Therefore, identifying novel molecular regulators remains important for improving our understanding and management of CCA.

Kinesin superfamily proteins are microtubule-dependent molecular motors that regulate intracellular transport of vesicles, organelles, and macromolecular complexes. The human kinesin superfamily comprises approximately 45 members classified into 14 families based on sequence homology and motor domain organization [7]. These proteins play essential roles in cell division, cytoskeletal organization, and signal transduction. Dysregulation of kinesin family members has been linked to tumor progression, metastasis, and therapeutic resistance across multiple cancer types [8,9,10]. The kinesin-1 complex consists of two kinesin heavy chains and two kinesin light chains. The heavy chains provide motor activity, whereas kinesin light chains mediate cargo recognition and adaptor binding, thereby regulating intracellular transport specificity [7,8].

Kinesin light chain 1 (KLC1) is a regulatory subunit of the kinesin-1 complex that mediates cargo binding through tetratricopeptide repeat domains. KLC1 interacts with adaptor proteins such as JNK-interacting protein (JIP) family members and calsyntenin-1 to facilitate microtubule-dependent transport of vesicles, mitochondria, and protein complexes [11,12,13]. In addition, phosphorylation of KLC1 regulates cargo binding and vesicular trafficking, suggesting that KLC1 may influence intracellular signaling by regulating the spatial distribution of transported molecules [14].

Emerging evidence indicates that kinesin light chain family members contribute to cancer progression. KLC2 promotes tumor cell proliferation, migration, and therapeutic resistance in lung cancer [15,16]. Similarly, KLC3 enhances ovarian cancer cell proliferation and migration through COL3A1-mediated activation of PI3K/AKT signaling [17], whereas KLC4 regulates radioresistance and tumor growth through mitochondrial dysfunction and apoptosis pathways in lung cancer [8,18]. Although studies on KLC1 remain limited, KLC1 has been identified as a fusion partner of ALK in lung cancer, producing a transforming KLC1-ALK fusion protein [19,20]. Moreover, kinesin-1 subunits, including KLC1, have been implicated in regulating epithelial–mesenchymal plasticity and tumor aggressiveness in breast cancer, suggesting that KLC1 may influence cell migration and invasion [21]. These findings support a potential role for KLC1 in regulating cancer-associated phenotypes.

In our previous proteomics study, treatment of CCA cells with 5′-deoxy-5′-methylthioadenosine (MTA) significantly reduced KLC1 protein expression and suppressed cell growth, migration, and invasion, suggesting that KLC1 may contribute to malignant phenotypes in CCA [22]. However, the functional significance and downstream molecular mechanisms of KLC1 in CCA remain unclear.

Therefore, the present study aimed to investigate the functional role of KLC1 in CCA using a loss-of-function approach. KLC1 expression was suppressed by siRNA-mediated knockdown, and its effects on cell growth, migration, and invasion were evaluated. To investigate molecular alterations associated with KLC1 silencing, transcriptomic profiling was performed using RNA sequencing, followed by functional enrichment and protein–protein interaction analysis. Representative genes from key pathways were subsequently validated by RT–qPCR to support the transcriptomic findings and further characterize signaling pathways associated with KLC1-mediated regulation of aggressive phenotypes in CCA cells.

## 2. Results

### 2.1. KLC1 Expression and Its Association with Clinicopathological Characteristics in CCA

**Figure 1:**
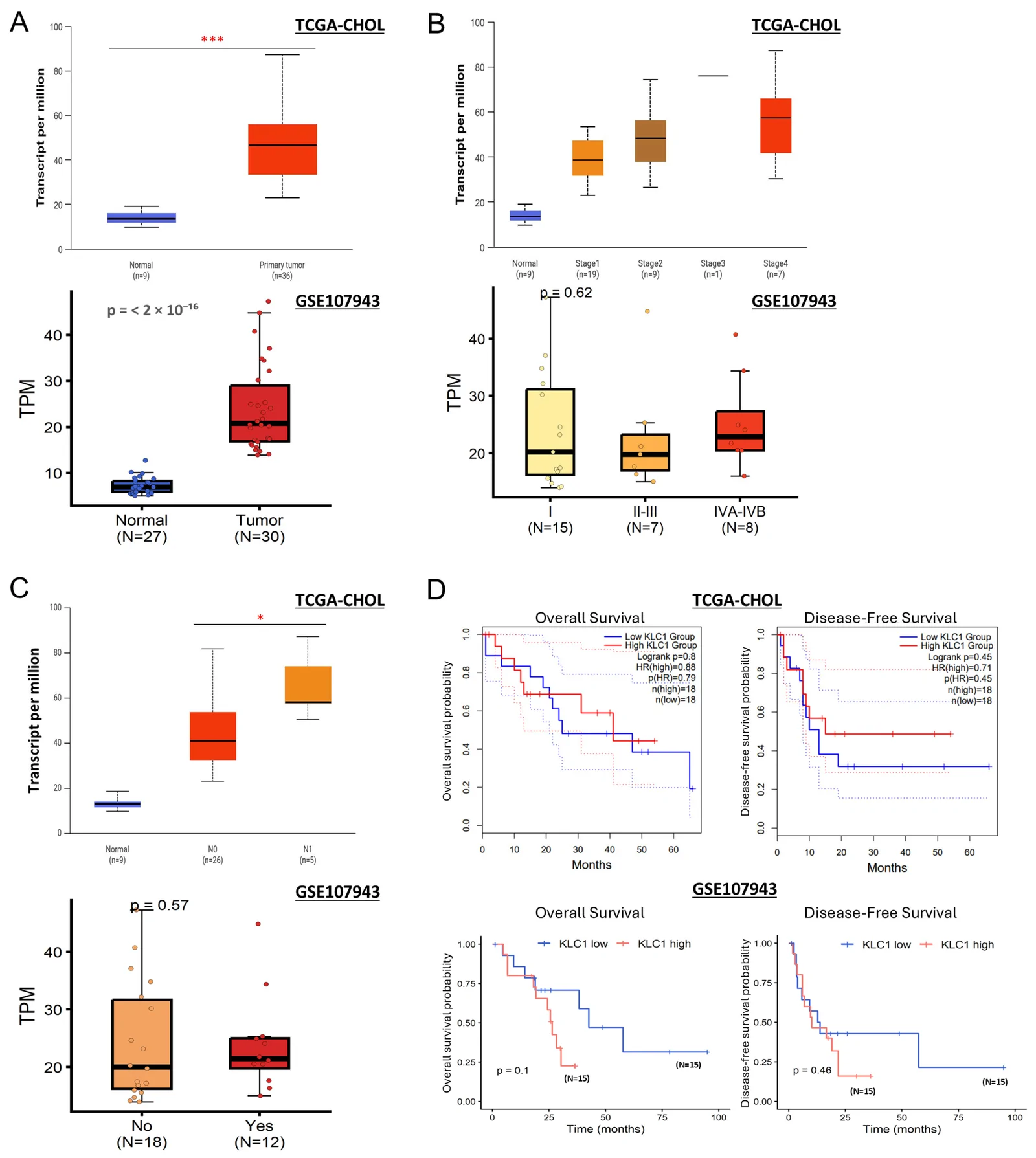
KLC1 expression and clinical implications in CCA. (**A**) KLC1 mRNA expression in CCA and non-tumor tissues from TCGA-CHOL and GSE107943 datasets. (**B**) Association between KLC1 expression and tumor stage. (**C**) Association between KLC1 expression and metastasis-related clinicopathological parameters. In TCGA-CHOL, N0 and N1 indicate lymph node-negative and lymph node-positive cases, respectively. In GSE107943, “No” and “Yes” indicate cases without and with vascular invasion, respectively. (**D**) Kaplan–Meier analysis of OS and DFS according to KLC1 expression levels. Upper and lower panels represent TCGA-CHOL and GSE107943 datasets, respectively. * *p* < 0.05, *** *p* < 0.001.

To assess the expression pattern of KLC1 in clinical CCA datasets, mRNA expression data were obtained from two independent public datasets, the TCGA-CHOL cohort and the GEO GSE107943 dataset. In both datasets, KLC1 expression was significantly upregulated in CCA tissues compared with non-tumor tissues (Figure 1A). When patients were stratified by tumor stage, an upward trend in KLC1 expression was observed in the TCGA-CHOL cohort; however, no significant differences were detected among stages in either dataset (Figure 1B). Regarding metastasis-associated clinicopathological features, KLC1 expression tended to be higher in the N1 (lymph node-positive) group in the TCGA-CHOL cohort, whereas no significant association was observed with vascular invasion status in the GSE107943 dataset (Figure 1C). In addition, survival analysis showed no significant association between KLC1 expression and OS or DFS (Figure 1D).

**Figure 2:**
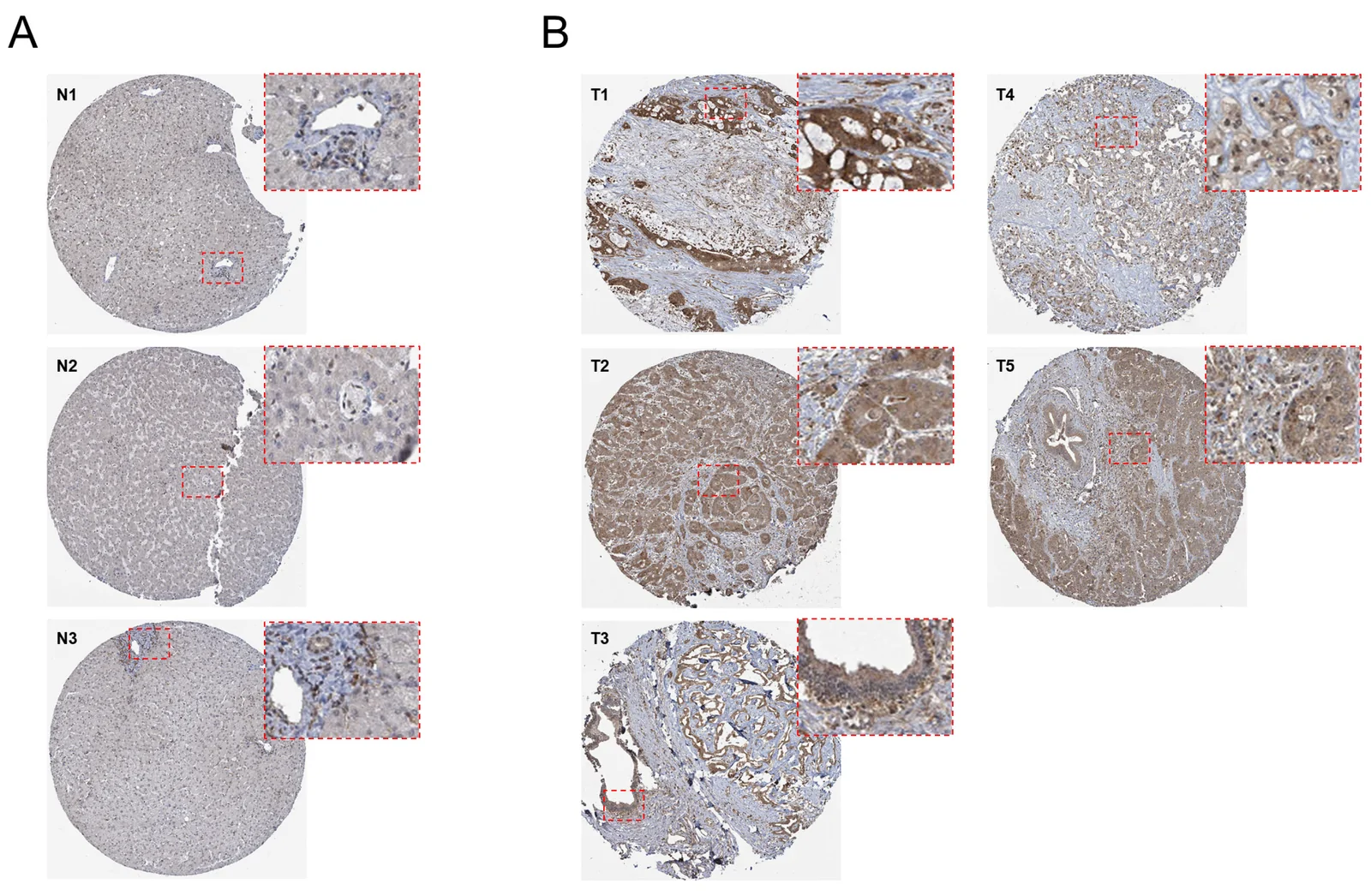
KLC1 protein expression in CCA tissues from the Human Protein Atlas (HPA). Representative immunohistochemistry (IHC) images of KLC1 in (**A**) normal liver tissues (*n* = 3) and (**B**) CCA tissues (*n* = 5) using the anti-KLC1 antibody (HPA044617). Red dashed boxes indicate regions shown at higher magnification in the corresponding insets.

KLC1 protein expression was further evaluated using the HPA database. IHC images from normal liver and CCA tissues were retrieved and compared. In normal liver tissues, KLC1 staining was generally weak in both hepatocytes and bile duct epithelial cells (Figure 2A). In contrast, CCA tissues generally exhibited stronger KLC1 immunoreactivity, with moderate to strong staining observed in all five tumor tissue samples (Figure 2B). Because the HPA data are based on a limited number of representative immunohistochemical images without quantitative scoring, these observations should be considered descriptive.

### 2.2. KLC1 Knockdown Suppresses Aggressive Phenotypes in CCA Cells

**Figure 3:**
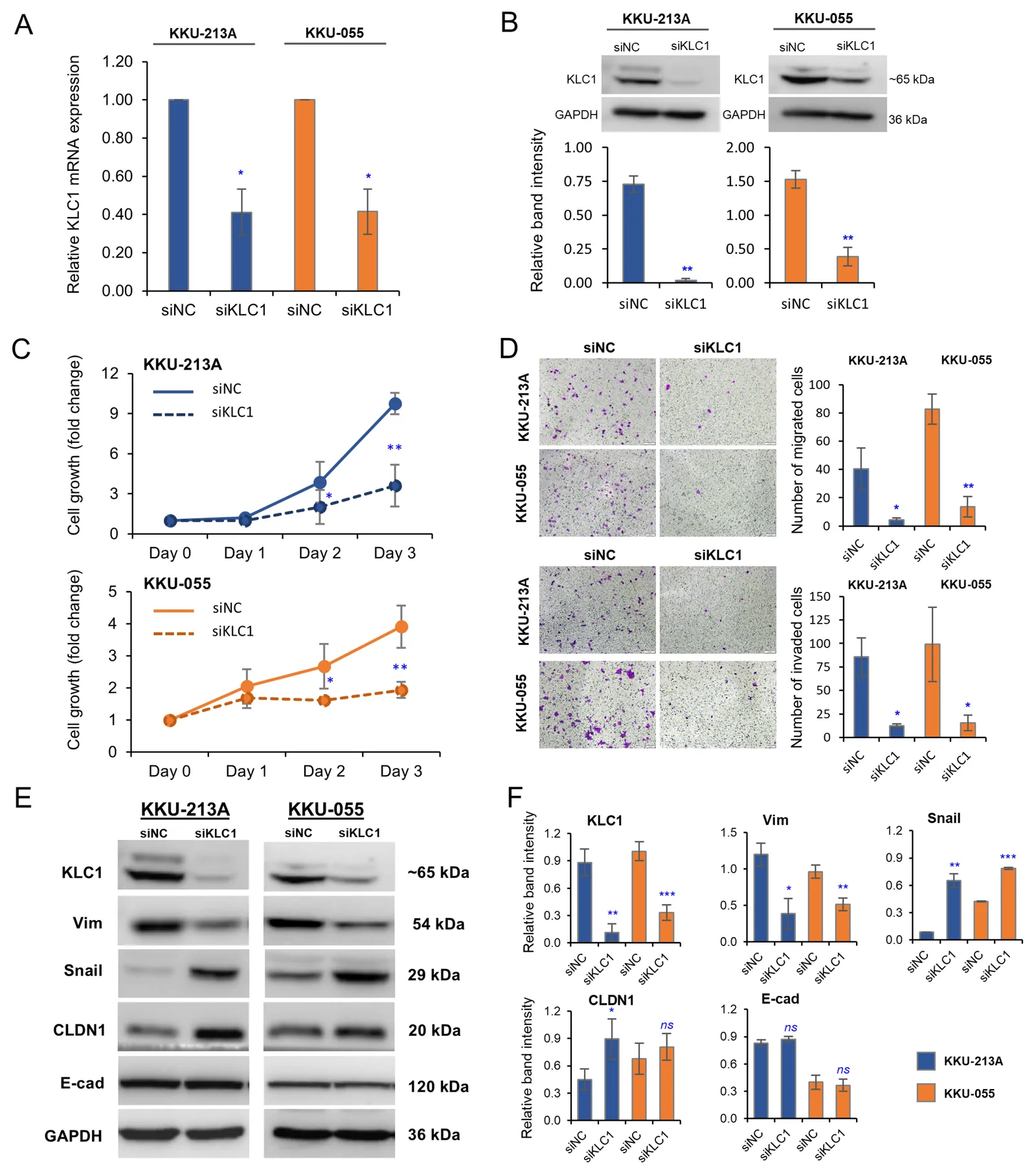
Effects of KLC1 knockdown on aggressive phenotypes of CCA cells. (**A**) KLC1 knockdown efficiency at the mRNA level determined by RT–qPCR. (**B**) KLC1 protein expression determined by Western blot analysis. (**C**) Cell growth following KLC1 knockdown assessed using the MTT assay. (**D**) Cell migration and invasion evaluated using modified Boyden chamber assays. (**E**) Expression of EMT-related proteins following KLC1 depletion analyzed by Western blotting. (**F**) Quantification of protein expression using Fiji (version 2.16.0; ImageJ 1.54p). Data are presented as the mean ± SD from three independent biological replicates (*n* = 3). * *p* < 0.05, ** *p* < 0.01, and *** *p* < 0.001 vs. siNC; ns, not significant.

To investigate the functional role of KLC1, loss-of-function experiments were performed using siRNA-mediated knockdown. Knockdown efficiency was first evaluated at the mRNA level. At 48 h after transfection, KLC1 mRNA expression was reduced by approximately 60% in both KKU-213A and KKU-055 cells (Figure 3A). Western blot analysis further demonstrated that KLC1 protein levels were markedly decreased, with reductions of >90% and approximately 70% in KKU-213A and KKU-055 cells, respectively (Figure 3B).

Cell viability was assessed using the MTT assay, and absorbance values were normalized to day 0 to evaluate relative cell growth. KLC1 knockdown significantly reduced relative cell growth compared with that in control cells. In KKU-213A cells, cell growth increased to 3.9-fold relative to day 0 in control cells versus to 2.0-fold in KLC1 knockdown cells on day 2 and to 9.8-fold versus to 3.6-fold on day 3, respectively. Similarly, in KKU-055 cells, cell growth increased to 2.7-fold relative to day 0 in control cells versus to 1.6-fold in KLC1 knockdown cells on day 2 and to 3.9-fold versus to 1.9-fold on day 3, respectively (Figure 3C). To determine whether differences in short-term cell viability could have influenced the migration and invasion assays, cell viability was assessed over the corresponding assay periods (12 h for KKU-213A and 15 h for KKU-055). No significant differences were observed between the siNC and siKLC1 groups in either cell line (Figure S1).

The effect of KLC1 depletion on cell motility was further evaluated using Transwell migration and invasion assays. KLC1 knockdown markedly reduced cell migration by approximately 89% in KKU-213A cells and 84% in KKU-055 cells. Consistently, KLC1 depletion also suppressed cell invasion, reducing the number of invaded cells by approximately 85% in KKU-213A cells and 84% in KKU-055 cells (Figure 3D).

### 2.3. KLC1 Knockdown Alters the Expression of EMT-Related Proteins

Because cell motility was suppressed following KLC1 knockdown, the expression of EMT-related proteins was further examined in KLC1-depleted cells. Four EMT markers, vimentin, Snail, claudin-1, and E-cadherin, were analyzed by Western blotting. KLC1 knockdown decreased vimentin expression, whereas claudin-1 expression was increased compared with control cells, although this increase was not significant in KKU-055 cells. In contrast, E-cadherin levels remained largely unchanged. Unexpectedly, Snail expression was increased following KLC1 knockdown (Figure 3E). Densitometric quantification confirmed these changes (Figure 3F). The raw data supporting the functional assays and Western blot quantification are provided in Table S1.

### 2.4. Transcriptomic Profiling Reveals Global Gene Expression Changes Following KLC1 Knockdown

**Figure 4:**
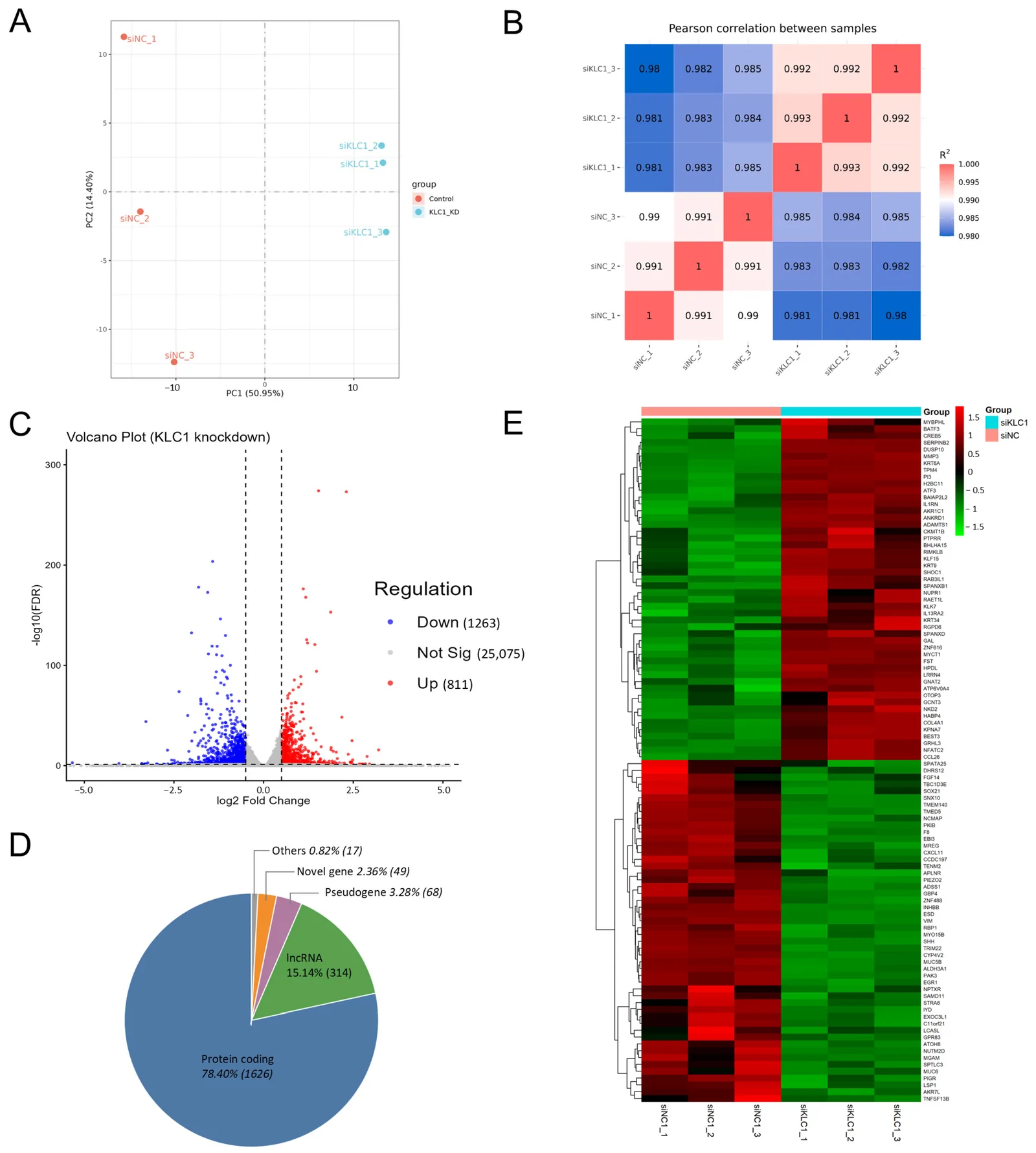
Transcriptomic analysis of KLC1 knockdown in CCA cells. (**A**) Principal component analysis (PCA) of siNC and siKLC1 samples based on global gene expression profiles. (**B**) Pearson correlation heatmap showing sample reproducibility among biological replicates. (**C**) Volcano plot showing differentially expressed genes (DEGs) following KLC1 knockdown. Red and blue dots represent upregulated and downregulated genes, respectively. (**D**) Classification of significant DEGs according to gene biotype. (**E**) Hierarchical clustering heatmap showing the top differentially expressed genes between siNC and siKLC1 samples.

To further investigate molecular changes induced by KLC1 depletion, transcriptomic profiling was performed using RNA sequencing in KKU-213A cells transfected with siNC or siKLC1. PCA demonstrated clear separation between siNC and siKLC1 samples along PC1 (50.95%) and PC2 (14.40%) (Figure 4A). Consistently, sample-to-sample correlation analysis demonstrated high reproducibility among biological replicates within each group (r = 0.980–1.00), whereas slightly lower correlations were observed between control and KLC1 knockdown samples (Figure 4B).

Differential expression analysis revealed widespread transcriptional alterations after KLC1 knockdown. Using a cutoff of FDR < 0.05 and |log2FC| ≥ 0.5, a total of 2074 genes were identified as significantly differentially expressed among 27,149 genes analyzed. Of these, 1263 genes were significantly downregulated and 811 genes were upregulated, whereas 25,075 genes showed no significant change (Figure 4C). Among the significant DEGs, 1626 were protein-coding genes, 314 were long non-coding RNAs (lncRNAs), 68 were pseudogenes, 49 were novel genes, and 17 belonged to other gene biotypes (Figure 4D). Heatmap visualization of the top 50 upregulated and top 50 downregulated genes revealed clear separation between control and KLC1 knockdown samples, with consistent clustering of biological replicates within each condition (Figure 4E). Detailed information for the genes displayed in the heatmap is provided in Table S2, whereas the complete list of differentially expressed genes is provided in Supplementary Table S3.

### 2.5. Functional Enrichment and Protein–Protein Interaction Analyses of KLC1-Regulated Genes

**Figure 5:**
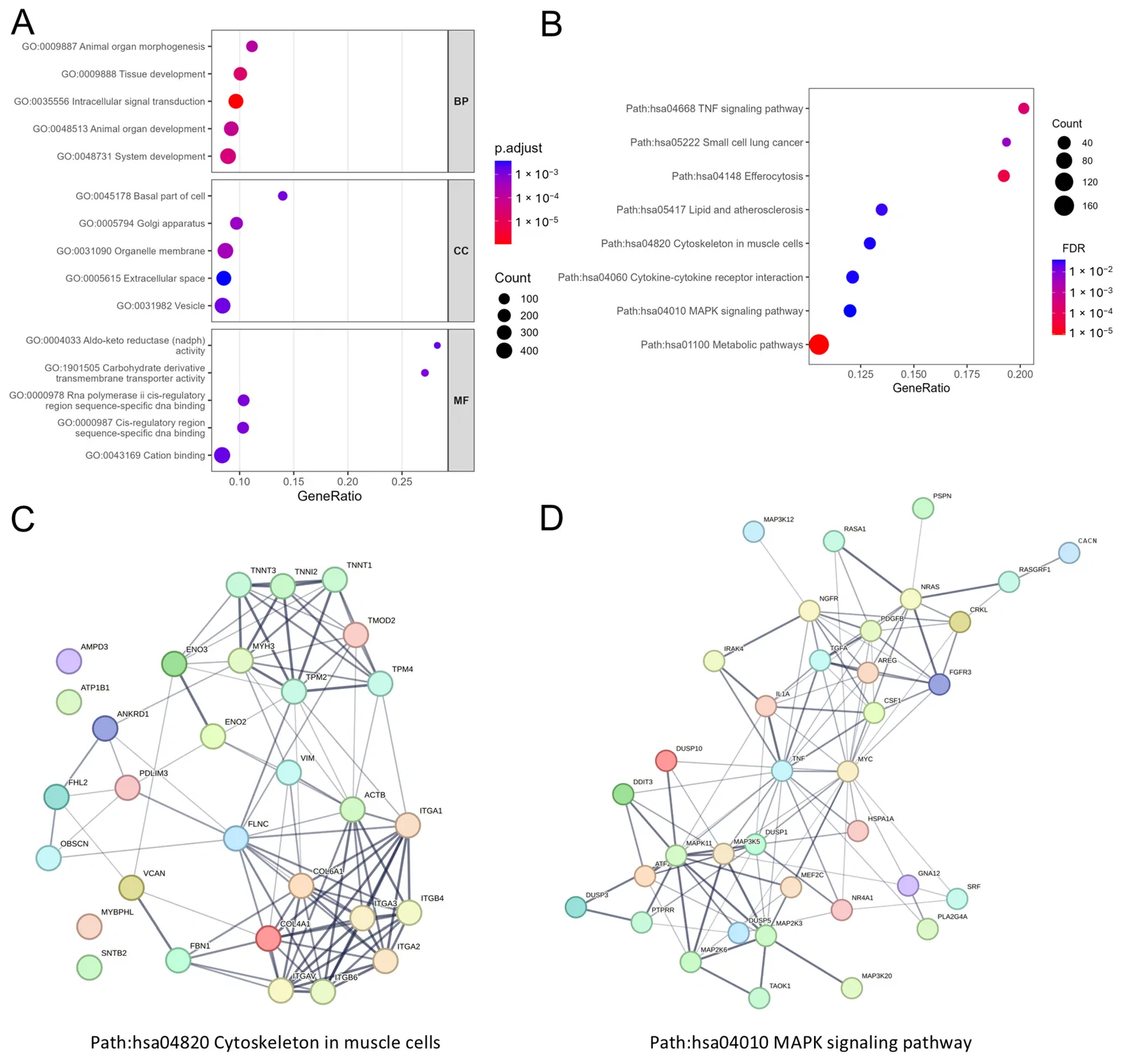
Functional enrichment and PPI analysis of KLC1-regulated DEGs. (**A**) GO enrichment analysis of protein-coding DEGs showing the top five terms for BP, CC, and MF categories. (**B**) KEGG pathway enrichment analysis showing significantly enriched pathways. Dot size represents gene counts, and color indicates adjusted *p*-values (FDR). (**C**) PPI network of DEGs enriched in “Cytoskeleton in muscle cells” (hsa04820). (**D**) PPI network of DEGs enriched in “MAPK signaling pathway” (hsa04010). PPI networks were generated using STRING version 12.0.

Functional enrichment analysis was performed using the 1626 significantly differentially expressed protein-coding genes to identify biological functions associated with KLC1 knockdown. GO analysis revealed 211 significantly enriched BP terms, of which the top five included animal organ morphogenesis, tissue development, intracellular signal transduction, animal organ development, and system development. In the CC category, 13 significant terms were identified, with the top five enriched terms comprising basal part of cell, Golgi apparatus, organelle membrane, extracellular space, and vesicle. For MF, 57 significant terms were detected, and the top five included aldo–keto reductase (NADPH) activity, carbohydrate derivative transmembrane transporter activity, RNA polymerase II cis-regulatory region sequence-specific DNA binding, cis-regulatory region sequence-specific DNA binding, and cation binding (Figure 5A). These enriched GO terms suggest that KLC1 knockdown affects multiple biological processes related to tissue development, intracellular signaling, subcellular organization, and transcriptional regulation. The top enriched GO terms are summarized in Table 1, whereas the complete GO enrichment results are provided in Table S4.

KEGG pathway analysis identified eight significantly enriched pathways, which are visualized in Figure 5B. The top enriched pathways are summarized in Table 2, whereas the complete GO and KEGG enrichment results are provided in Table S4. These pathways included metabolic pathways, the MAPK signaling pathway, cytokine–cytokine receptor interaction, cytoskeleton in muscle cells, lipid and atherosclerosis, efferocytosis, small cell lung cancer, and the TNF signaling pathway. These findings indicate that KLC1 knockdown is associated with transcriptomic alterations in metabolic processes, cytokine-associated signaling, cytoskeletal organization, and cancer-related signaling pathways.

To further investigate the functional relationships among genes within KLC1 knockdown, two significantly enriched KEGG pathways, “Cytoskeleton in muscle cells” (hsa04820) and “MAPK signaling pathway” (hsa04010), were selected for PPI network analysis based on their enrichment significance and the presence of multiple differentially expressed genes within each pathway. These pathways were further prioritized because they included genes associated with cell proliferation, migration, and invasion, which are consistent with the phenotypic changes observed following KLC1 depletion.

The PPI network generated from the cytoskeleton-related pathway revealed a densely interconnected module consisting of extracellular matrix components, integrins, and cytoskeletal regulators (Figure 5C). Many genes within this network are associated with structural organization and cell motility. Based on network connectivity and differential expression patterns, representative genes were selected for validation. Downregulated genes included COL6A1, ITGA3, ITGAV, and VIM, whereas ANKRD1, TPM4, SNAI1, and VCAN were selected as upregulated genes. These genes were chosen because they are involved in cytoskeletal organization, extracellular matrix remodeling, and adhesion-related processes associated with cell migration and invasion.

Similarly, the PPI network constructed from the MAPK signaling pathway showed a connected module composed of kinases, transcription factors, and feedback regulators (Figure 5D). This network included several signaling components associated with proliferation and stress response pathways. Representative genes were selected from both downregulated and upregulated groups. Downregulated genes included ATF2, FGFR3, MAP2K6, MAPK11, and NRAS, while upregulated genes included DDIT3, DUSP1, DUSP5, and NR4A1. These genes were selected because they represent differentially expressed components of the enriched MAPK signaling pathway identified by KEGG analysis.

### 2.6. Validation of Selected Genes by RT–qPCR

To validate RNA-seq findings, representative genes from the cytoskeleton and MAPK signaling pathways were analyzed by RT–qPCR in KKU-213A and KKU-055 cells. In the cytoskeleton-related pathway, KKU-213A cells showed decreased expression of COL6A1, ITGA3, ITGAV, and VIM following KLC1 knockdown, whereas ANKRD1, TPM4, SNAI1, and VCAN were upregulated compared with control cells. Similar expression patterns were observed in KKU-055 cells for most selected genes, except that COL6A1 was upregulated and ANKRD1 expression remained unchanged, further supporting the involvement of KLC1 in regulating cytoskeleton- and extracellular matrix-associated genes.

**Figure 6:**
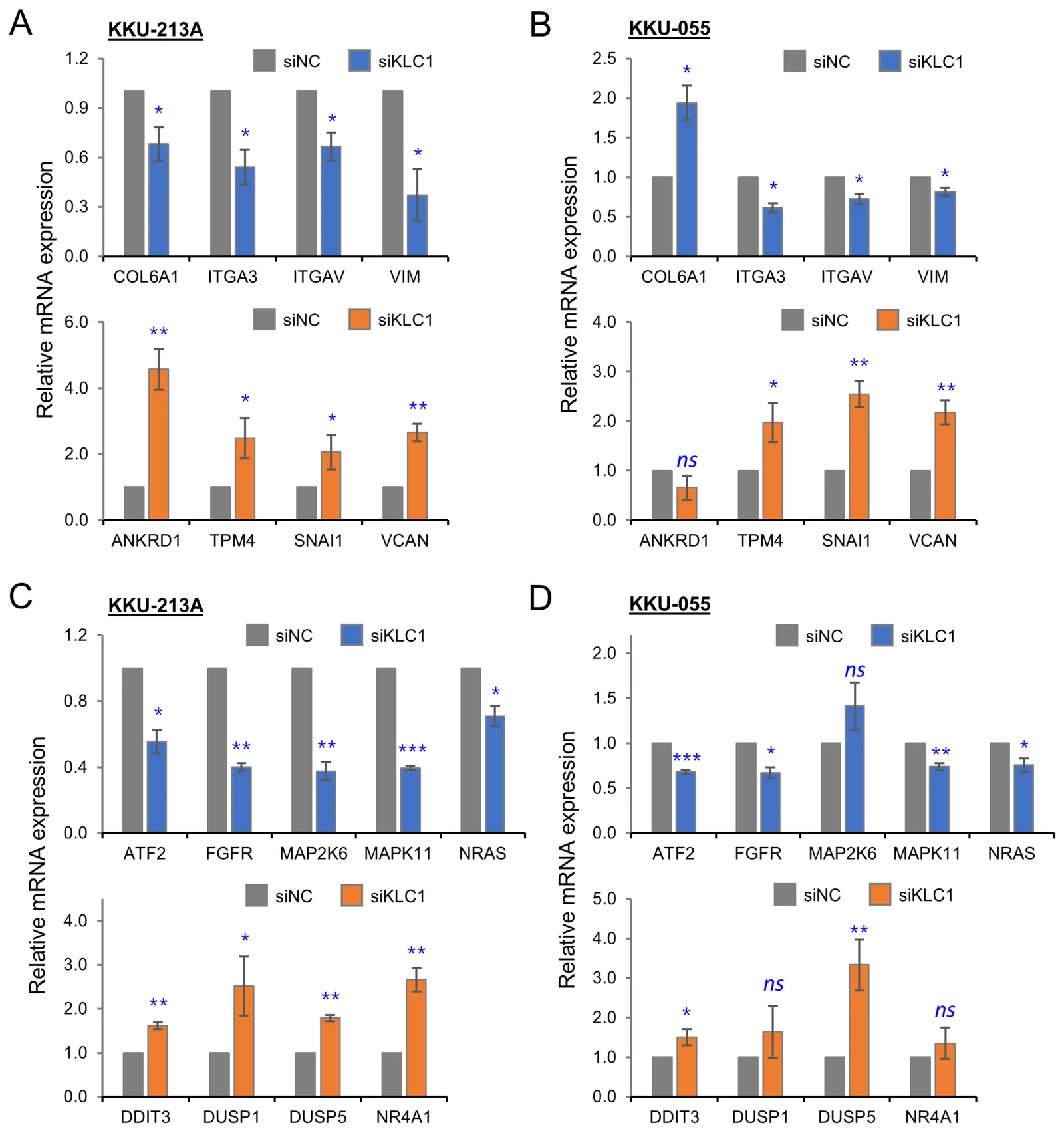
Validation of selected DEGs following KLC1 knockdown by RT–qPCR. Expression of selected genes from the “Cytoskeleton in muscle cells” pathway in (**A**) KKU-213A and (**B**) KKU-055 cells, and selected genes from the “MAPK signaling pathway” in (**C**) KKU-213A and (**D**) KKU-055 cells. Gray bars represent siNC, whereas colored bars represent siKLC1. Genes selected from the downregulated and upregulated RNA-seq datasets are indicated by the corresponding colors. Data are presented as the mean ± SD from three independent biological replicates (*n* = 3). * *p* < 0.05, ** *p* < 0.01, and *** *p* < 0.001 vs. siNC; ns, not significant.

For the MAPK signaling pathway, KKU-213A cells showed decreased expression of ATF2, FGFR3, MAP2K6, MAPK11, and NRAS, together with increased expression of DDIT3, DUSP1, DUSP5, and NR4A1 following KLC1 depletion. In KKU-055 cells, ATF2, FGFR3, MAPK11, and NRAS were also significantly downregulated following KLC1 knockdown, whereas MAP2K6 exhibited a non-significant increasing trend. Among the upregulated genes, DDIT3 and DUSP5 showed expression patterns consistent with those observed in KKU-213A cells, whereas DUSP1 and NR4A1 also exhibited increasing trends that did not reach statistical significance. Overall, the RT–qPCR results were largely consistent with the RNA-seq data, supporting the reliability of the transcriptomic findings and confirming the involvement of cytoskeleton-associated and MAPK signaling pathways in KLC1-mediated regulation of CCA cells (Figure 6A–D). The raw RT–qPCR data are provided in Table S5.

## 3. Discussion

In the present study, we explored the functional role of KLC1 in CCA and demonstrated that KLC1 contributes to aggressive tumor phenotypes. Analysis of public datasets revealed that KLC1 expression was elevated in CCA tissues compared with non-tumor tissues, and immunohistochemical data further supported stronger protein expression in tumor cells. Although KLC1 expression was not significantly associated with patient survival, its increased expression in tumor tissues together with a trend toward higher expression in advanced clinicopathological features, suggests that KLC1 may be involved in tumor progression rather than serving as an independent prognostic marker. This pattern is consistent with reports on other kinesin superfamily members, in which elevated expression correlates with tumor aggressiveness but not always with survival outcomes [23]. These observations suggest that KLC1 may function as a regulator of malignant phenotypes in CCA.

Loss-of-function experiments further supported this hypothesis. siRNA-mediated knockdown of KLC1 significantly suppressed cell growth, migration, and invasion in both KKU-213A and KKU-055 cells. Importantly, KLC1 knockdown did not significantly affect cell growth during the 12-h (KKU-213A) or 15-h (KKU-055) Transwell assay periods, suggesting that the observed reductions in migration and invasion are unlikely to be attributable to short-term differences in cell growth. The consistent effects observed across two independent CCA cell lines support a potential role for KLC1 in promoting aggressive tumor behavior. Similar oncogenic roles have been reported for other kinesin light chain family members; for example, KLC3 knockdown suppressed proliferation, migration, and epithelial–mesenchymal transition in ovarian cancer cells in vitro and inhibited tumor growth in vivo [17], while KLC2 overexpression promoted cell proliferation and clonogenic potential in chronic myeloid leukemia [24].

KLC1 functions as an adaptor subunit of the kinesin-1 motor complex that mediates the transport of diverse cellular cargoes along microtubules. KLC1 associates with kinesin heavy chains to form the kinesin-1 complex, whereas its tetratricopeptide repeat (TPR) domains mediate interactions with cargo adaptor proteins involved in the trafficking of vesicles, mitochondria, and Golgi-derived compartments [12,13]. The TPR domains of KLC1 have been shown to recruit multiple scaffold proteins, including c-Jun N-terminal kinase (JNK)-interacting protein 1 (JIP1) and JNK-associated leucine zipper protein (JLP), thereby coordinating the spatial organization of signaling complexes along microtubule tracks [25,26]. Through these adaptor functions, KLC1 regulates intracellular cargo transport and facilitates the spatial organization of signaling complexes.

Given that kinesin light chains regulate cargo transport and cytoskeletal organization, depletion of KLC1 may disrupt intracellular trafficking and structural dynamics required for tumor cell growth and motility [9,27]. These findings are also consistent with our previous proteomics study, in which MTA treatment reduced KLC1 protein expression and suppressed CCA cell aggressiveness, and in which TCGA data confirmed upregulation of KLC1 mRNA in CCA tumor tissues relative to adjacent non-tumor tissues, further supporting a functional role for KLC1 in tumor progression [22].

Consistent with the reduced migratory and invasive abilities observed following KLC1 knockdown, changes in EMT-related proteins were also detected. Vimentin expression was decreased, whereas claudin-1 and Snail were increased, while E-cadherin remained largely unchanged. Interestingly, although Snail expression was increased following KLC1 knockdown, cell migration and invasion were markedly suppressed. Because EMT is regulated by multiple interconnected signaling pathways and transcription factors, increased Snail expression alone may not be sufficient to drive a mesenchymal phenotype. This expression pattern suggests that KLC1 depletion induces remodeling of epithelial–mesenchymal characteristics rather than a complete reversal of EMT. The relationship between KLC1 and EMT-related regulation appears to be context-dependent. In breast cancer, KLC1 has been reported to regulate epithelial–mesenchymal plasticity and influence invasive behavior through kinesin-1-associated mechanisms [21]. Therefore, the role of KLC1 in regulating EMT-associated processes may depend on tumor type and cellular context. Accordingly, the conclusions of the present study are limited to CCA, and whether KLC1 exerts similar or distinct functions in other cancer types requires further investigation. The coexistence of epithelial and mesenchymal features may reflect partial EMT or epithelial–mesenchymal plasticity, which has been increasingly recognized as an intermediate state associated with altered motility and cytoskeletal organization. These findings indicate that KLC1 may influence cell motility through modulation of cytoskeletal and adhesion-related components.

To further understand the molecular mechanisms underlying these phenotypic changes, transcriptomic profiling was performed following KLC1 knockdown. RNA sequencing revealed widespread transcriptional alterations, with 2074 genes significantly differentially expressed. Among these, 1626 protein-coding genes were subjected to functional enrichment analysis. GO analysis indicated enrichment in biological processes related to development, intracellular signaling, and membrane organization, whereas KEGG pathway analysis identified multiple significantly enriched pathways, including MAPK signaling and cytoskeleton-related pathways. These pathways were particularly notable because they were consistent with the observed phenotypic changes in cell growth, migration, and invasion. Kinesin superfamily proteins have been broadly implicated in the regulation of multiple oncogenic signaling networks, including MAPK, PI3K/Akt, and Wnt/β-catenin pathways across diverse cancer types [10,28].

Protein–protein interaction network analysis further highlighted interconnected gene modules within these pathways. The cytoskeleton-related pathway formed a densely connected network consisting of extracellular matrix components, integrins, and cytoskeletal regulators. Several genes within this network, including COL6A1, ITGA3, ITGAV, and VIM, were downregulated following KLC1 knockdown, whereas ANKRD1, TPM4, SNAI1, and VCAN were upregulated. These genes are involved in extracellular matrix organization, adhesion, and cytoskeletal remodeling, suggesting that KLC1 depletion disrupts structural components required for cell migration and invasion. Comparable cytoskeletal remodeling was reported in ovarian cancer following KLC3 knockdown, where collagen-related downstream genes cooperated with kinesin light chain signaling to sustain malignant phenotypes [17]. The coordinated alteration in these genes is consistent with the reduced motility observed in functional assays.

Similarly, the MAPK signaling pathway revealed coordinated changes in signaling regulators associated with cell growth and stress responses. Downregulated genes included ATF2, FGFR3, MAP2K6, MAPK11, and NRAS, whereas DDIT3, DUSP1, DUSP5, and NR4A1 were upregulated. These genes participate in MAPK-mediated transcriptional regulation and cellular signaling. Importantly, KLC1-mediated cargo transport has been reported to be dynamically regulated by phosphorylation. Specifically, serine 460 of KLC1 has been identified as a target of extracellular signal-regulated kinase (ERK), and phosphorylation at this site modulates the binding and trafficking of calsyntenin-1, a vesicular cargo adaptor protein [14]. This regulatory mechanism supports the possibility that KLC1 may influence MAPK-associated signaling through spatial control of signaling components. Phosphorylation-dependent regulation of kinesin light chain cargo binding has also been reported for KLC2, where glycogen synthase kinase-3β-mediated phosphorylation controls the release of Smad2 cargo, linking kinesin-1 transport to TGF-β signaling [29]. Scaffold proteins such as JIP-1b have similarly been shown to bridge KLC1 to cargo receptors and to modulate downstream JNK-mediated phosphorylation events, underscoring the broader role of KLC1 in coordinating signaling pathway activities [11].

The simultaneous downregulation of growth-associated components and upregulation of negative regulators suggest alterations in MAPK-associated signaling networks following KLC1 depletion [14]. Such changes may contribute to the reduced cell growth observed in KLC1 knockdown cells. This interpretation is further supported by broader evidence that kinesin superfamily proteins regulate key oncogenic signaling pathways in cancer, and their dysregulation can alter tumor cell proliferative capacity [9,27].

RT–qPCR validation largely confirmed the expression patterns of selected genes from both cytoskeleton-associated and MAPK signaling pathways. The majority of transcriptomic alterations identified in KKU-213A cells were further validated in KKU-055 cells. The consistency between RNA-seq and validation experiments supports the robustness of the transcriptomic analysis and reinforces the involvement of cytoskeletal remodeling and MAPK signaling in KLC1-mediated regulation. The use of an independent CCA cell line further strengthens the reproducibility of these findings, consistent with methodological approaches used in other kinesin light chain studies [8,18].

Beyond the present CCA context, KLC1 has been identified as a fusion partner in non-small cell lung cancer (NSCLC), where KLC1–ALK gene fusions have been described as oncogenic drivers [20]. Clinical cases involving KLC1–ALK rearrangements have demonstrated variable sensitivity to ALK tyrosine kinase inhibitors, including instances of primary resistance, highlighting the biological complexity of KLC1–ALK fusion-driven tumors [30,31]. Although these findings underscore the clinical significance of KLC1-associated genomic rearrangements in NSCLC, the present study did not investigate KLC1–ALK fusions, and therefore no conclusions regarding their relevance to cholangiocarcinoma can be drawn from our data. Future studies are warranted to determine whether KLC1–ALK rearrangements or related mechanisms are involved in CCA.

Several limitations of this study should be acknowledged. Functional analyses were performed in vitro using two CCA cell lines, and in vivo validation is required to confirm the role of KLC1 in tumor growth and metastasis, as demonstrated in xenograft models for other kinesin family members [8,17]. In addition, the clinical analyses were based on publicly available datasets with relatively small patient cohorts, and the HPA protein expression data were derived from a limited number of representative IHC images without quantitative scoring. Therefore, these findings should be considered descriptive, and larger clinical studies with quantitative protein expression analyses are required to determine the diagnostic, prognostic, and therapeutic relevance of KLC1 in CCA. Furthermore, siRNA-mediated knockdown provides only transient gene suppression. In the present study, the functional analyses were performed using a single KLC1-targeting siRNA. Although KLC1 knockdown was confirmed at both the mRNA and protein levels, a non-targeting siRNA was included as a negative control, and consistent phenotypic effects were observed in two independent CCA cell lines, the use of a single targeting sequence cannot entirely exclude sequence-specific off-target effects. Therefore, complementary approaches such as additional independent KLC1-targeting siRNAs, stable knockdown, siRNA-resistant rescue experiments, or overexpression models would further clarify the mechanistic role of KLC1. Identification of direct cargo molecules transported by KLC1 may also provide additional insight into downstream signaling mechanisms, given that multiple cargo–adaptor interactions have been characterized for KLC1 in other cellular contexts [12,14,26].

## 4. Materials and Methods

### 4.1. Cell Lines and Cell Culture

Two human CCA cell lines, namely KKU-213A and KKU-055, established from primary intrahepatic CCA tissues of Thai patients with opisthorchiasis-associated CCA, were obtained from the Japanese Collection of Research Bioresources (JCRB) Cell Bank, Ibaraki, Osaka, Japan. The identities of both cell lines were authenticated by the JCRB Cell Bank prior to distribution. All cells were maintained in high-glucose Dulbecco’s Modified Eagle Medium (DMEM) supplemented with 10% fetal bovine serum (FBS) and 1% antibiotics/antimycotics. The cells were cultured under standard conditions in a humidified incubator at 37 °C with 5% CO_2_. All experiments were performed using cells between passages 20 and 30. Cells were routinely observed for morphology, maintained under aseptic conditions, and tested for mycoplasma contamination before each series of experiments and at least every 3 months. All cultures used in this study were confirmed to be mycoplasma-free. All experiments were performed using cells in the logarithmic growth phase.

### 4.2. siRNA Transfection

Small interfering RNA (siRNA) targeting KLC1 and a negative control siRNA were purchased from Gene Universal Inc. The sequences were as follows: KLC1 siRNA, sense 5′-AUAACCUGGCAUCCUGCUATT-3′ and antisense 5′-UAGCAGGAUGCCAGGUUAUTT-3′; negative control siRNA (siNC), sense 5′-UUCUCCGAACGUGUCACGUTT-3′ and antisense 5′-ACGUGACACGUUCGGAGAATT-3′. CCA cells at a density of 1 × 10^5^ cells/well were seeded in 6-well plates and incubated overnight prior to transfection. The medium was then replaced with serum-free Opti-MEM (Thermo Fisher Scientific, Waltham, MA, USA) and incubated for approximately 1 h. The transfection complex, composed of KLC1 siRNA or siNC at a final concentration of 100 nM and Lipofectamine™ 2000 (Invitrogen, Thermo Fisher Scientific, Vilnius, Lithuania), was prepared and incubated for 15 min before being gently added dropwise to each well, followed by gentle tilting of the plate. The 100 nM siRNA concentration was selected based on our established laboratory protocol, which consistently achieves efficient gene silencing in CCA cells. Transfection efficiency was confirmed by the marked reduction in KLC1 expression at both the mRNA and protein levels. After 6 h, the transfection medium was removed and replaced with complete DMEM, and cells were further incubated for 48 h before being harvested for protein and RNA extraction. For phenotypic studies, cells were incubated for 24 h post-transfection, then trypsinized and used for cell growth, migration, and invasion assays.

### 4.3. RNA Extraction and RT–qPCR

Total RNA was extracted from cells using TRIzol reagent (Invitrogen, Thermo Fisher Scientific, Vilnius, Lithuania) according to the manufacturer’s instructions. The concentration and quality of RNA were assessed using a Spark multimode microplate reader (Tecan Group Ltd., Männedorf, Switzerland) and agarose gel electrophoresis. Complementary DNA (cDNA) was synthesized using the RevertAid First Strand cDNA Synthesis Kit (Thermo Fisher Scientific, Vilnius, Lithuania) according to the manufacturer’s protocol.

Quantitative real-time PCR (qPCR) was performed using the QuantiNova SYBR Green PCR Kit (Qiagen, Hilden, Germany) on a QuantStudio™ 3 Real-Time PCR System (Applied Biosystems, Waltham, MA, USA). The thermocycling conditions were as follows: initial denaturation at 95 °C for 15 min; followed by 35 cycles of denaturation at 95 °C for 20 s, annealing at 55–60 °C for 1 min, and extension at 72 °C for 2 min. Melt curve analysis was performed at 95 °C for 15 s, 60 °C for 1 min, and 95 °C for 15 s to verify amplification specificity. A single peak was observed for each primer pair, indicating specific amplification without detectable primer-dimer formation. A no-template control (NTC) and a no-reverse transcriptase (no-RT) control were included in each qPCR run, and no amplification was detected in either control. Glyceraldehyde-3-phosphate dehydrogenase (GAPDH) was used as the internal reference gene. The expression levels of KLC1 and other target genes were quantified using gene-specific primers listed in Table 3, which includes primer sequences and amplicon sizes. For each independent biological replicate, technical triplicate Cq values were averaged prior to analysis. Relative gene expression was calculated using the 2^−ΔΔCq^ method for graphical presentation, whereas statistical analyses were performed using ΔCq values. RT–qPCR analyses were performed using three independent biological replicates, each measured in technical triplicate.

### 4.4. Western Blot Analysis

Total protein was extracted from cells using RIPA lysis buffer (Visual Protein, Taipei, Taiwan) supplemented with phosphatase and protease inhibitor cocktail (Roche Diagnostics GmbH, Mannheim, Germany). Cell lysates were centrifuged at 15,000× *g* for 20 min at 4 °C, and protein concentration was determined using a BCA protein assay kit (Bio Basic Inc., Markham, ON, Canada). Equal amounts of protein (30 µg per lane) were separated by 12% SDS–PAGE and transferred onto PVDF membranes (Cytiva, Freiburg, Germany) using a Trans-Blot^®^ TurboTM transfer system (Bio-Rad, Hercules, CA, USA). Membranes were blocked with 2% skim milk in TBS containing 0.1% Tween-20 (TBS-T) for 1 h at room temperature and incubated with primary antibodies overnight at 4 °C. After washing, membranes were incubated with HRP-conjugated secondary antibodies (Table 4) for 1 h at room temperature. Protein bands were detected using SuperSignal™ West Pico PLUS Chemiluminescent Substrate (Thermo Fisher Scientific, Waltham, MA, USA) and visualized using a GelDoc Alliance Atom Q9 imaging system (Uvitec, Cambridge, UK). Densitometric analysis was performed using Fiji (version 2.16.0; ImageJ 1.54p, National Institutes of Health, Bethesda, MD, USA) from the original uncropped, non-saturated chemiluminescent images. All images were converted to 8-bit grayscale before analysis. For each blot, identical rectangular regions of interest (ROIs) were applied to all lanes. Local background intensity was measured from an adjacent band-free region using an ROI of identical size and subtracted from the raw integrated density (RawIntDen) of each band to obtain the background-corrected intensity. The background-corrected intensity of each target protein was normalized to the corresponding background-corrected GAPDH intensity from the same lane to obtain the normalized protein expression. Normalized intensity values from three independent biological experiments were used for statistical analysis.

### 4.5. MTT Assay

Cell viability was assessed using the MTT assay as a metabolic activity-based proxy for viable cell number. KKU-213A and KKU-055 cells were transfected with siNC or siKLC1 for 24 h, then seeded into 96-well plates at a density of 1.5 × 10^3^ cells per well in DMEM containing 2% FBS. Cells were cultured under standard conditions and assessed at 6, 24, 48, and 72 h post-seeding. The 6 h time point, representing cell attachment without significant proliferation, was used as the baseline (day 0). At each indicated time point, MTT reagent [3-(4,5-dimethylthiazol-2-yl)-2,5-diphenyltetrazolium bromide] was added to a final concentration of 0.5 mg/mL and incubated for 2 h at 37 °C. The medium was then removed, and the resulting formazan crystals were dissolved in 100 µL dimethyl sulfoxide (DMSO). Absorbance was measured at 540 nm using a Spark multimode microplate reader (Tecan Group Ltd., Männedorf, Switzerland). Absorbance values were normalized to day 0 and expressed as fold changes over time to evaluate relative cell growth. All measurements were performed in triplicate, and experiments were independently repeated.

### 4.6. Cell Migration and Invasion Assays

The migratory and invasive capacities of CCA cells were assessed using modified Boyden chamber assays. KKU-213A and KKU-055 cells were first subjected to siRNA transfection (siNC or siKLC1) for 24 h. Subsequently, cells were collected, resuspended in serum-free DMEM, and seeded into the upper compartments of Transwell inserts (8 µm pore size; Corning, NY, USA) at a density of 3 × 10^4^ cells in 200 µL. The lower chambers were filled with 600 µL DMEM containing 10% FBS as a chemoattractant.

Following incubation at 37 °C in a humidified atmosphere with 5% CO_2_ for 12 h (KKU-213A) and 15 h (KKU-055), non-migrated cells on the upper surface of the membrane were carefully removed using cotton swabs. Cells that migrated to the underside of the membrane were fixed with 4% paraformaldehyde at room temperature and stained with 0.4% sulforhodamine B (SRB). After washing to remove excess dye, stained cells were visualized and counted under a light microscope (Olympus CKX53, Tokyo, Japan) in three randomly selected fields per insert.

For invasion assays, the upper surface of the inserts was pre-coated with Matrigel (Corning, NY, USA) and allowed to polymerize at 37 °C prior to cell seeding. The remaining procedures were performed as described for the migration assay.

### 4.7. Public Database Analysis of KLC1 Expression and Clinical Significance

Publicly available datasets were used to investigate the expression profile and clinical relevance of KLC1 in CCA. KLC1 mRNA expression in normal and tumor tissues, as well as its association with clinicopathological features, including tumor stage, lymph node status, and patient survival, were analyzed using The Cancer Genome Atlas cholangiocarcinoma (TCGA-CHOL) cohort via the University of Alabama at Birmingham Cancer Data Analysis Portal (UALCAN) [32]. Subgroup analyses were performed based on tumor stage and lymph node status (N0 vs. N1). Kaplan–Meier survival analyses for overall survival (OS) and disease-free survival (DFS) were conducted using the Gene Expression Profiling Interactive Analysis 2 (GEPIA2) web server [33].

To validate these results, an independent dataset (GSE107943) from the Gene Expression Omnibus (GEO) was analyzed. KLC1 mRNA expression and clinicopathological data were processed using R (version 4.5.2). Comparisons between adjacent non-tumor and tumor tissues and stratification by tumor stage and vascular invasion (“No” vs. “Yes”) were performed. Survival analyses (OS and DFS) were conducted by dividing patients into high- and low-KLC1 expression groups based on the median value.

KLC1 protein expression was further assessed using immunohistochemical (IHC) data from the Human Protein Atlas (HPA). Representative images stained with anti-KLC1 antibody (HPA044617) were compared between normal (*n* = 3) and tumor tissues (*n* = 5).

### 4.8. RNA Sequencing

Total RNA was extracted from KKU-213A cells transfected with either KLC1-targeting siRNA (siKLC1) or negative control siRNA (siNC) 48 h after transfection, with three biological replicates per group. RNA samples were submitted to Novogene AIT Genomics Singapore Pte. Ltd. (Singapore) for strand-specific mRNA sequencing library preparation and high-throughput sequencing. Prior to library construction, RNA quality and integrity were assessed, and only high-quality RNA samples with RNA integrity number (RIN) values ranging from 9.1 to 9.3 were used for subsequent processing.

For library preparation, mRNA was enriched from total RNA using poly-T oligo-attached magnetic beads and fragmented into short fragments. The fragmented mRNA was reverse-transcribed for first-strand cDNA synthesis, followed by strand-specific second-strand cDNA synthesis. The resulting cDNA was subjected to end repair, adapter ligation, size selection, amplification, and purification to generate sequencing libraries. Library quality and fragment size distribution were evaluated using an Agilent 2100 Bioanalyzer (Agilent Technologies Inc., Santa Clara, CA, USA), and library concentrations were quantified by qPCR. Qualified libraries were sequenced on the Illumina NovaSeq X Plus (Illumina, Inc., San Diego, CA, USA) platform using a paired-end 150 bp (PE150) sequencing strategy. Raw sequencing reads were processed to remove adapter sequences, low-quality reads, and reads containing excessive unknown bases, generating high-quality clean reads for downstream transcriptomic analysis.

### 4.9. Differential Gene Expression and Functional Enrichment Analysis

Clean reads were aligned to the human reference genome (GRCh38) using HISAT2 (version 2.2.2), followed by transcript assembly and expression quantification using StringTie (version 3.0.3). Gene expression abundance was calculated as fragments per kilobase of transcript per million mapped reads (FPKM), while raw read counts were used for differential expression analysis. Differentially expressed genes (DEGs) between siKLC1 and siNC groups were identified using the DESeq2 package in R (version 4.5.2). Genes with a false discovery rate (FDR) < 0.05 and an absolute log_2_ fold change (|log2FC|) ≥ 0.5 were considered statistically significant. Variance-stabilizing transformation (VST) was applied to normalized count data for visualization and clustering analyses. Principal component analysis (PCA) and Pearson correlation analysis were performed to evaluate transcriptomic differences between experimental groups and reproducibility among biological replicates. Heatmaps and volcano plots were generated using the pheatmap and ggplot2 packages in R.

Functional enrichment analyses were performed using ShinyGO (version 0.85.1) [34] based on protein-coding DEGs. Gene Ontology (GO) enrichment analysis was conducted across biological process (BP), cellular component (CC), and molecular function (MF) categories. Kyoto Encyclopedia of Genes and Genomes (KEGG) pathway enrichment analysis was performed to identify biological pathways associated with KLC1 silencing. Enriched GO terms and KEGG pathways with FDR < 0.05 were considered statistically significant, and enrichment results were visualized using R.

### 4.10. Protein–Protein Interaction Network Analysis

Protein–protein interaction (PPI) network analysis was performed using the STRING database (version 12.0; https://string-db.org/ (accessed on 28 February 2026)) [35]. Differentially expressed genes involved in two significantly enriched KEGG pathways, “Cytoskeleton in muscle cells” (hsa04820) and “MAPK signaling pathway” (hsa04010), were selected for network analysis. Gene lists were uploaded to STRING, with *Homo sapiens* selected as the reference organism. The PPI networks were constructed using the full STRING network with a minimum required interaction score of 0.400 (medium confidence). Network-associated functional annotations were analyzed using the built-in STRING enrichment tools, and enriched terms with an FDR < 0.05 were considered statistically significant.

### 4.11. Statistical Analysis

All experiments were performed with at least three independent biological replicates, and data are presented as the mean ± standard deviation (SD). Statistical analyses were performed using R (version 4.5.2). Comparisons between two groups were analyzed using a paired two-tailed Student’s *t*-test. A *p* < 0.05 was considered to indicate a statistically significant difference.

For RNA sequencing and functional enrichment analyses, statistical methods and significance criteria were applied as described in the corresponding sections. Multiple testing correction was performed using the Benjamini–Hochberg method to control the false discovery rate (FDR). For public database analyses, comparisons between two independent groups were evaluated using the two-sided Wilcoxon rank-sum test, whereas comparisons among multiple groups were performed using the Kruskal–Wallis test. Survival analyses were performed using the Kaplan–Meier method, and differences between groups were evaluated using the two-sided log-rank test.

## 5. Conclusions

**Figure 7:**
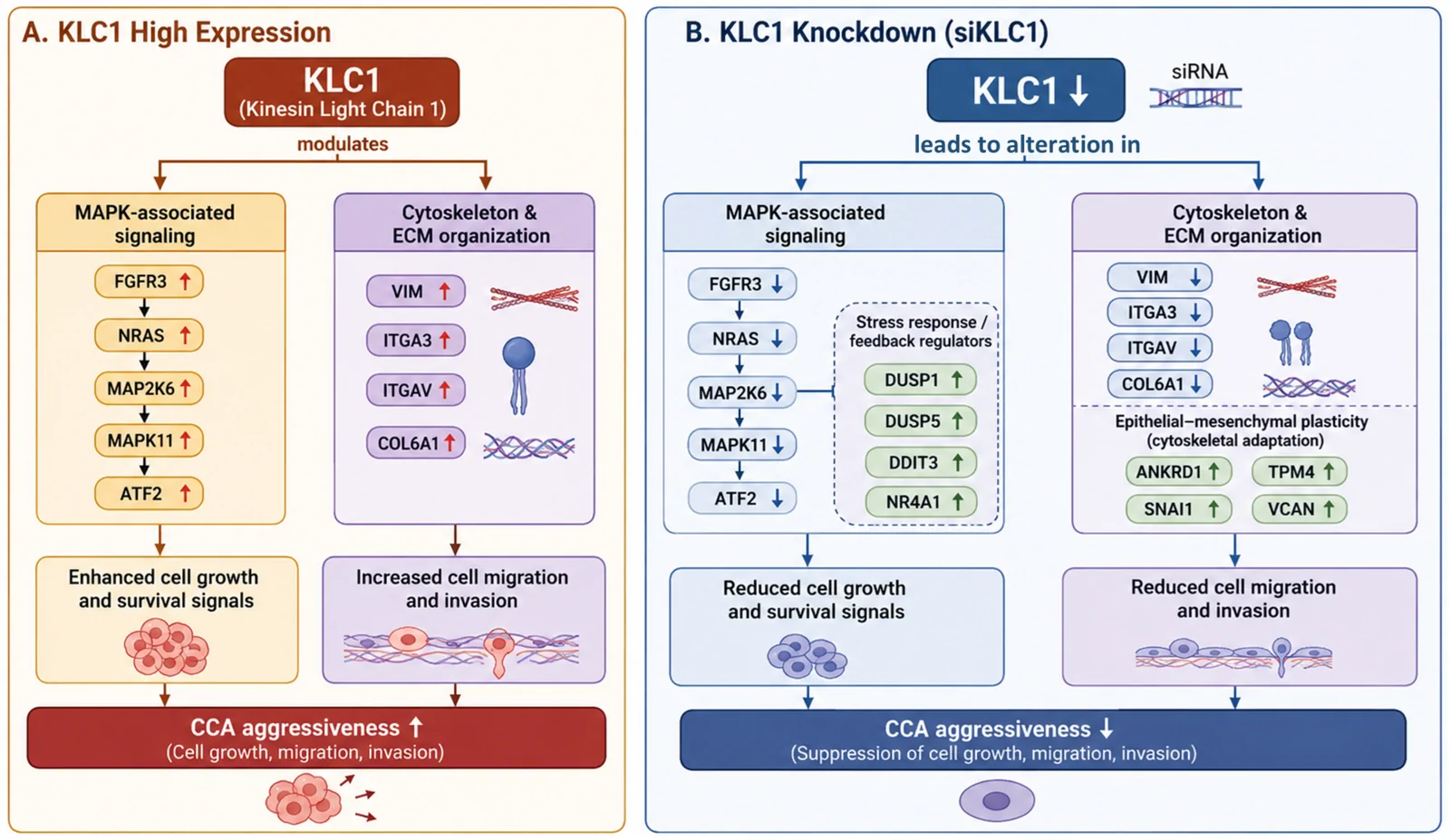
Proposed mechanism of KLC1-mediated regulation of CCA progression. (**A**) Under conditions of high KLC1 expression, KLC1 contributes to aggressive phenotypes of CCA cells through modulation of MAPK-associated signaling and cytoskeleton/extracellular matrix (ECM)-related pathways, resulting in enhanced cell growth, migration, and invasion. (**B**) KLC1 knockdown alters MAPK-associated signaling and cytoskeleton/ECM-related gene networks, leading to the suppression of aggressive phenotypes in CCA cells. Upward (↑) and downward (↓) arrows indicate increased and decreased expression, respectively.

This study demonstrates that KLC1 contributes to aggressive phenotypes in CCA cells. KLC1 knockdown significantly suppressed cell growth, migration, and invasion and induced widespread transcriptional alterations, particularly in genes associated with cytoskeletal organization and MAPK signaling. These findings suggest that KLC1 may contribute to aggressive phenotypes in CCA, at least in part, through transcriptomic alterations involving cytoskeletal organization and MAPK-associated signaling. Collectively, this study provides new insights into the biological role of KLC1 in CCA and highlights KLC1 as a candidate for further investigation as a potential therapeutic target. Further studies are warranted to elucidate the underlying molecular mechanisms and validate its functional and therapeutic significance in vivo. A schematic summary of the proposed mechanism is presented in Figure 7.

## Figures and Tables

**Table 1:** Top enriched GO terms.

Ontology	Enriched Term	Gene Count	Fold Enrichment	Adjusted *p* Value (FDR)
Biological Process	Animal organ morphogenesis	121	1.612	1.58 × 10^−4^
	Tissue development	218	1.456	1.99 × 10^−5^
	Intracellular signal transduction	313	1.397	1.01 × 10^−6^
	Animal organ development	298	1.335	8.46 × 10^−5^
	System development	383	1.292	3.26 × 10^−5^
Cellular Component	Basal part of cell	46	2.022	0.00123
	Golgi apparatus	183	1.405	5.30 × 10^−4^
	Organelle membrane	377	1.256	3.53 × 10^−4^
	Extracellular space	309	1.235	0.00381
	Vesicle	385	1.218	0.00149
Molecular Function	Aldo-keto reductase (NADPH) activity	13	4.088	0.00192
	Carbohydrate derivative transmembrane transporter activity	16	3.922	0.00107
	RNA polymerase II cis-regulatory region sequence-specific DNA binding	131	1.500	0.00107
	Cis-regulatory region sequence-specific DNA binding	133	1.492	0.00107
	Cation binding	415	1.212	0.00171

**Table 2:** Top enriched KEGG pathways.

Enriched Pathway	Gene Count	Fold Enrichment	Adjusted *p* Value (FDR)
TNF signaling pathway	24	2.917	1.99 × 10^−4^
Small cell lung cancer	18	2.799	5.05 × 10^−3^
Efferocytosis	30	2.782	4.78 × 10^−5^
Lipid and atherosclerosis	29	1.951	2.91 × 10^−2^
Cytoskeleton in muscle cells	30	1.753	3.63 × 10^−2^
Cytokine–cytokine receptor interaction	36	1.870	3.63 × 10^−2^
MAPK signaling pathway	36	1.736	3.82 × 10^−2^

**Table 3:** Primer sequences and amplicon sizes used for RT–qPCR.

Gene		Sequence (5′–3′)	Amplicon Size (bp)
*GAPDH*	For:	CAAATTCCATGGCACCGTCA	132
	Rev:	GACTCCACGACGTACTCAG	
*KLC1*	For:	TGAGGTCTCGTAAACAGGGT	167
	Rev:	AAGATCAGTGCCATCTTCCTCC	
*ANKRD1*	For:	CGCCCGAGATAAGTTGCTCA	94
	Rev:	GTCTGCCTCACAGGCGATAA	
*ATF2*	For:	AAACTGCACAGCCCACATCA	143
	Rev:	TGGTGCCTGGGTGATTACAG	
*COL6A1*	For:	GGCTCAGACCAGCTCAATGT	129
	Rev:	GGCGGTGACATTCTTCAGGA	
*DDIT3*	For:	ATGAACGGCTCAAGCAGGAA	148
	Rev:	GGGAAAGGTGGGTAGTGTGG	
*DUSP1*	For:	GGATACGAAGCGTTTTCGGC	91
	Rev:	GGACGCTAGTACTCAGGGGA	
*DUSP5*	For:	CCAGCTCCTGCAGTACGAAT	145
	Rev:	GAATGTGCAGTAGGCACCCT	
*FGFR3*	For:	TATTTATGGGCCCCTGGCAC	128
	Rev:	TCCCTCTATCGGCGGGATAA	
*ITGA3*	For:	GCGCAAGGAGTGGGACTTAT	95
	Rev:	TGAAGCTGCCTACCTGCATC	
*ITGAV*	For:	CTGCCTGGAACAGCTCTCAA	150
	Rev:	CAGTGCTCGTCGAATTGCTC	
*MAP2K6*	For:	ACAAGTCCTGCCAAGTGGTT	125
	Rev:	AGGAGGGACAAACGCAGAAG	
*MAPK11*	For:	CACATGTGATGTGGTGCGTG	117
	Rev:	GCATCAGGAACCGAGGAGAG	
*NR4A1*	For:	CCAGCATTATGGTGTCCGCA	105
	Rev:	ACAGGGCAGTCCTTGTTAGC	
*NRAS*	For:	CTCAGCCAAGACCAGACAGG	117
	Rev:	CCCATACAACCCTGAGTCCC	
*SNAI1*	For:	TCTCTTCCTTGGAGGCCGA	174
	Rev:	GGCTTCGGATGTGCATCTTG	
*TPM4*	For:	TCTCTGGAGGCTGCATCTGA	112
	Rev:	CTGCAAATTCAGCACGGGTC	
*VIM*	For:	GGACCAGCTAACCAACGACA	178
	Rev:	AAGGTCAAGACGTGCCAGAG	
*VCAN*	For:	ATCGCTGCAAAATGAACCCG	99
	Rev:	ACTGGTCTCCGCTGTATCCT	

**Table 4:** List of antibodies.

Antibody	Host	Catalog Number	Dilution	Manufacturer
Anti-KLC1	Rabbit	ab174273	1:10,000	Abcam
Anti-Vimentin	Rabbit	5741	1:5000	Cell Signaling
Anti-Snail	Rabbit	3879	1:5000	Cell Signaling
Anti-Claudin-1	Rabbit	13255	1:5000	Cell Signaling
Anti-E-cadherin	Rabbit	3195	1:5000	Cell Signaling
Anti-GAPDH	Mouse	60004-1	1:10,000	Proteintech
HRP-linked anti-rabbit IgG	Goat	ab6721	1:10,000	Abcam
HRP-linked anti-mouse IgG	Goat	ab6789	1:10,000	Abcam

## Data Availability

All data supporting the findings of this study are available within the article and its Supplementary Materials. The RNA sequencing data generated in this study have been deposited in the Gene Expression Omnibus (GEO) database under accession number GSE337630.

## Supplementary Materials

The following supporting information can be downloaded at: https://www.mdpi.com/article/10.3390/ijms27177525/s1.

## Abbreviations

The following abbreviations are used in this manuscript:

ANOVA	Analysis of variance
ANKRD1	Ankyrin repeat domain 1
ATF2	Activating transcription factor 2
BP	Biological process
CC	Cellular component
CCA	Cholangiocarcinoma
CDK4/6	Cyclin-dependent kinase 4/6
cDNA	Complementary DNA
COL6A1	Collagen type VI alpha 1 chain
DDIT3	DNA damage inducible transcript 3
DMEM	Dulbecco’s Modified Eagle Medium
DMSO	Dimethyl sulfoxide
DUSP1	Dual specificity phosphatase 1
DUSP5	Dual specificity phosphatase 5
EMT	Epithelial–mesenchymal transition
FBS	Fetal bovine serum
FDR	False discovery rate
FGFR3	Fibroblast growth factor receptor 3
FPKM	Fragments per kilobase of transcript per million mapped reads
GAPDH	Glyceraldehyde-3-phosphate dehydrogenase
GEO	Gene Expression Omnibus
GO	Gene Ontology
HPA	Human Protein Atlas
IHC	Immunohistochemistry
ITGA3	Integrin subunit alpha 3
ITGAV	Integrin subunit alpha V
KEGG	Kyoto Encyclopedia of Genes and Genomes
KLC1	Kinesin light chain 1
MAP2K6	Mitogen-activated protein kinase kinase 6
MAPK	Mitogen-activated protein kinase
MAPK11	Mitogen-activated protein kinase 11
MTT	3-(4,5-Dimethylthiazol-2-yl)-2,5-diphenyltetrazolium bromide
NR4A1	Nuclear receptor subfamily 4 group A member 1
NRAS	NRAS proto-oncogene, GTPase
OS	Overall survival
PCA	Principal component analysis
PPI	Protein–protein interaction
qPCR	Quantitative polymerase chain reaction
RT–qPCR	Reverse transcription–quantitative polymerase chain reaction
SD	Standard deviation
SNAI1	Snail family transcriptional repressor 1
siRNA	Small interfering RNA
siNC	Negative control siRNA
TCGA	The Cancer Genome Atlas
TPM4	Tropomyosin 4
VCAN	Versican
VIM	Vimentin
VST	Variance-stabilizing transformation
