# Supplementary material for: Silencing of Kinesin Light Chain 1 Suppresses Aggressive Phenotypes in Cholangiocarcinoma Cells Through Transcriptomic Alterations
Source: Int J Mol Sci. 2026 Aug 22;27(17):7525. doi: 10.3390/ijms27177525 (PMC13566592; doi:10.3390/ijms27177525)
Supplement: Supplementary file 1 [file ijms-27-07525-s001.zip › Supplementary/Figure S1.pdf]

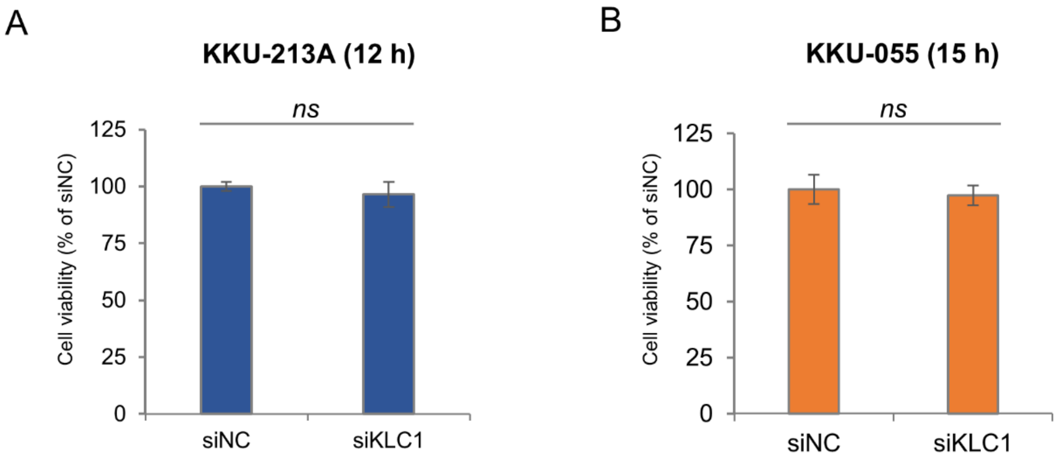

Supplementary Figure S1. KLC1 knockdown does not affect short-term cell viability during the Transwell assay period. Cell viability was measured 12 h after seeding in KKU-213A cells and 15 h after seeding in KKU-055 cells, corresponding to the durations of the migration and invasion assays. Cell viability was normalized to the mean value of the siNC group (100%). Data are presented as mean ± SD from three independent biological experiments. Statistical significance was determined using an unpaired two-tailed Student's t-test. No significant differences were observed between the siNC and siKLC1 groups (P = 0.358 for KKU-213A; P = 0.603 for KKU-055).
