# Supplementary material for: Silencing of Kinesin Light Chain 1 Suppresses Aggressive Phenotypes in Cholangiocarcinoma Cells Through Transcriptomic Alterations
Source: Int J Mol Sci. 2026 Aug 22;27(17):7525. doi: 10.3390/ijms27177525 (PMC13566592; doi:10.3390/ijms27177525)
Supplement: Supplementary file 1 [file ijms-27-07525-s001.zip › Supplementary/Figure S2.pdf]

Original Western blot images for Figure 3B.

KKU-213A

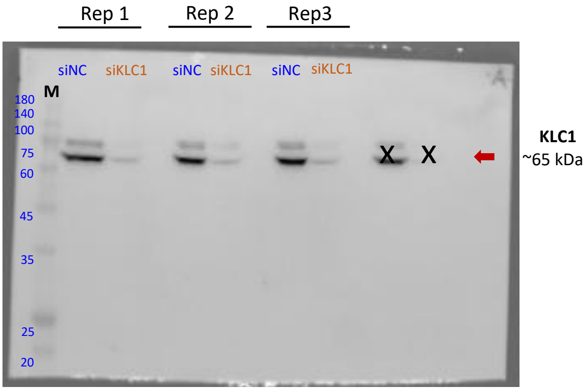

KLC1 protein detection: Total protein (30 µg/lane) from siKLC1-transfected KKU-213A cells was analyzed by Western blotting using rabbit anti-KLC1 (1:5,000; Abcam) and HRP-conjugated goat anti-rabbit IgG (1:10,000; Abcam). Exposure time : 1 min 30 sec.

KKU-055

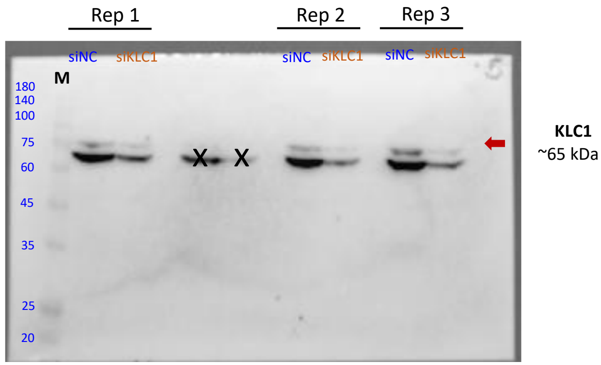

KLC1 protein detection: Total protein (30 µg/lane) from siKLC1-transfected KKU-055 cells was analyzed by Western blotting using rabbit anti-KLC1 (1:5,000; Abcam) and HRP-conjugated goat anti-rabbit IgG (1:10,000; Abcam). Exposure time : 3 min

KKU-213A

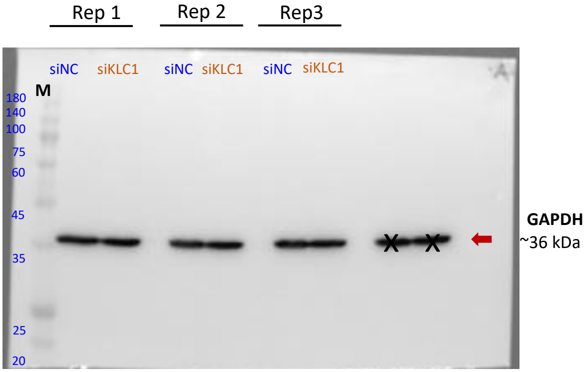

GAPDH protein detection: Total protein (30 µg/lane) from siKLC1-transfected KKU-213A cells was analyzed by Western blotting using mouse anti-GAPDH (1:10,000; Proteintech) and HRP-conjugated goat anti-mouse IgG (1:10,000; Abcam). Exposure time : 500 msec

KKU-055

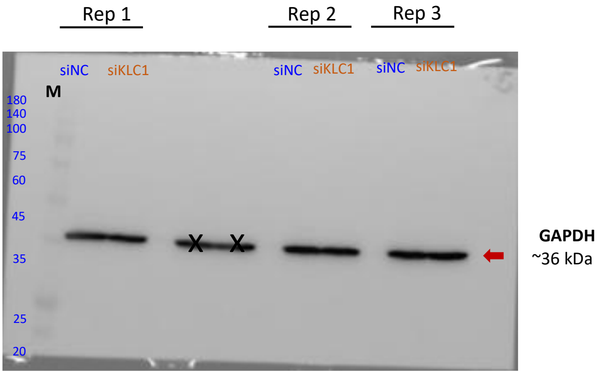

GAPDH protein detection: Total protein (30 µg/lane) from siKLC1-transfected KKU-055 cells was analyzed by Western blotting using mouse anti-GAPDH (1:10,000; Proteintech) and HRP-conjugated goat anti-mouse IgG (1:10,000; Abcam). Exposure time : 500 msec

Original Western blot images for Figure 3E.

KKU-213A Rep 1 Rep 2 Rep 3

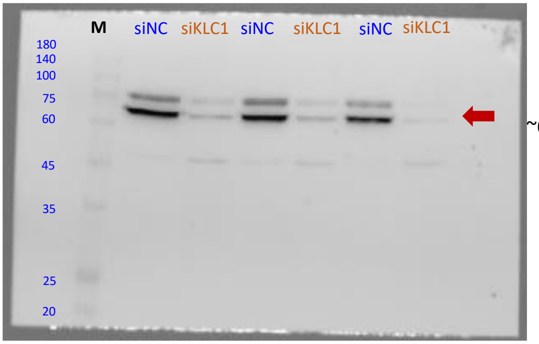

KLC1
~65 kDa

KLC1 protein detection: Total protein (30 µg/lane) from siKLC1-transfected KKU-213A cells was analyzed by Western blotting using rabbit anti-KLC1 (1:5,000; Abcam) and HRP-conjugated goat anti-rabbit IgG (1:10,000; Abcam). Exposure time : 1 min 15 sec 200 msec

KKU-055 Rep 1 Rep 2 Rep 3

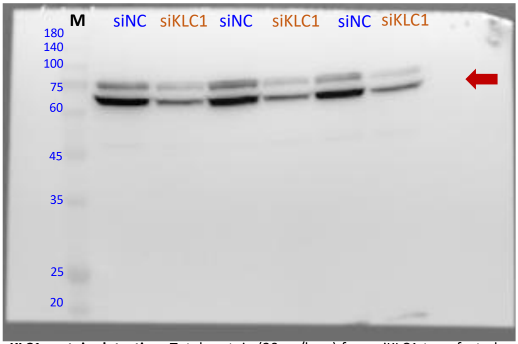

KLC1
~65 kDa

KLC1 protein detection: Total protein (30 µg/lane) from siKLC1-transfected KKU-055 cells was analyzed by Western blotting using rabbit anti-KLC1 (1:5,000; Abcam) and HRP-conjugated goat anti-rabbit IgG (1:10,000; Abcam). Exposure time : 40 sec 200 msec

KKU-213A Rep 1 Rep 2 Rep 3

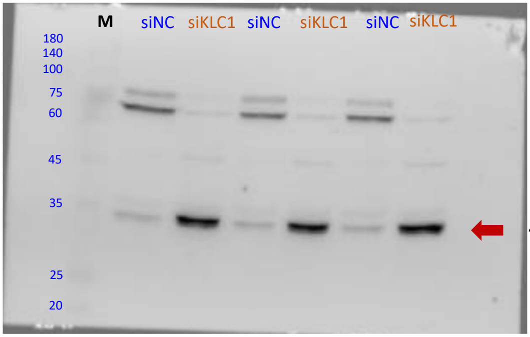

Snail
~29 kDa

Snail protein detection: Total protein (30 µg/lane) from siKLC1-transfected KKU-213A cells was analyzed by Western blotting using rabbit anti-Snail (1:5,000; Cell Signaling) and HRP-conjugated goat anti-rabbit IgG (1:10,000; Abcam). Exposure time : 1 min 30 sec 200 msec

KKU-055 Rep 1 Rep 2 Rep 3

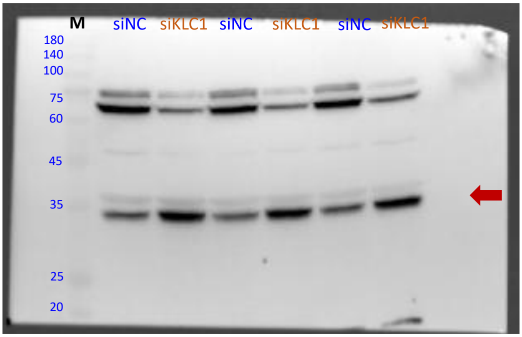

Snail
~29 kDa

Snail protein detection: Total protein (30 µg/lane) from siKLC1-transfected KKU-055 cells was analyzed by Western blotting using rabbit anti-Snail (1:5,000; Cell Signaling) and HRP-conjugated goat anti-rabbit IgG (1:10,000; Abcam). Exposure time : 2 min 00 sec 300 msec

KKU-213A Rep 1 Rep 2 Rep 3

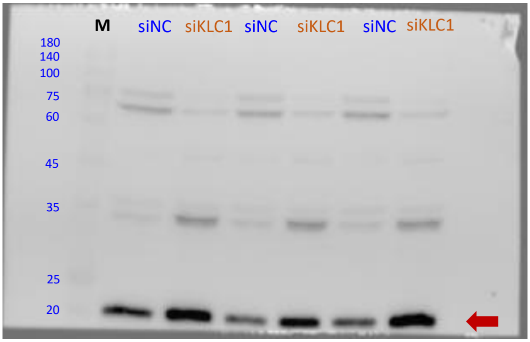

Claudin-1
~20 kDa

Claudin-1 protein detection: Total protein (30 µg/lane) from siKLC1-transfected KKU-213A cells was analyzed by Western blotting using rabbit anti-claudin-1 (1:5,000; Cell Signaling) and HRP-conjugated goat anti-rabbit IgG (1:10,000; Abcam). Exposure time : 30 sec 100 msec

KKU-055 Rep 1 Rep 2 Rep 3

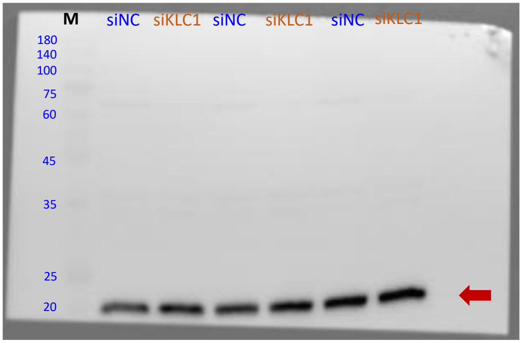

Claudin-1
~20 kDa

Claudin-1 protein detection: Total protein (30 µg/lane) from siKLC1-transfected KKU-055 cells was analyzed by Western blotting using rabbit anti-claudin-1 (1:5,000; Cell Signaling) and HRP-conjugated goat anti-rabbit IgG (1:10,000; Abcam). Exposure time : 1 sec 100 msec

Original Western blot images for Figure 3E.

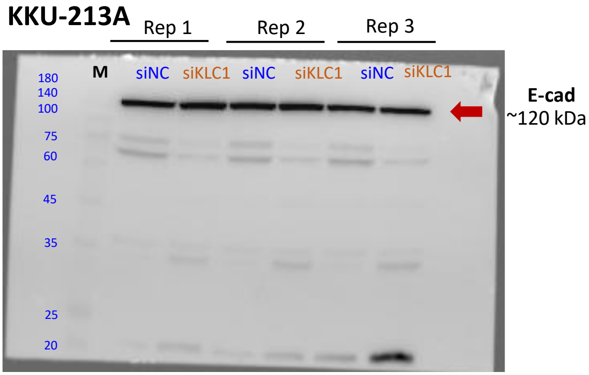

E-cadherin protein detection: Total protein (30 µg/lane) from siKLC1-transfected KKU-213A cells was analyzed by Western blotting using rabbit anti-E-cadherin (1:5,000; Cell Signaling) and HRP-conjugated goat anti-rabbit IgG (1:10,000; Abcam). Exposure time : 20 sec 200 msec

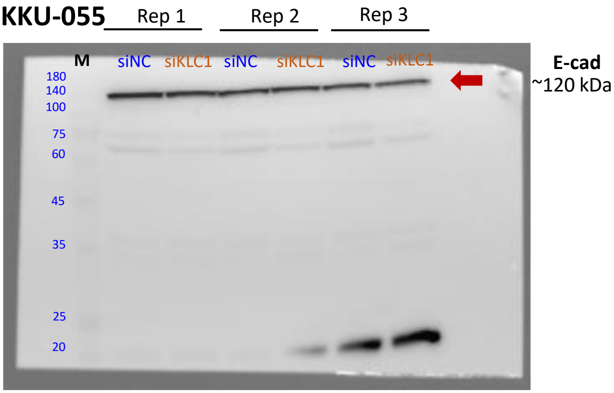

E-cadherin protein detection: Total protein (30 µg/lane) from siKLC1-transfected KKU-055 cells was analyzed by Western blotting using rabbit anti-E-cadherin (1:5,000; Cell Signaling) and HRP-conjugated goat anti-rabbit IgG (1:10,000; Abcam). Exposure time : 5 sec 100 msec

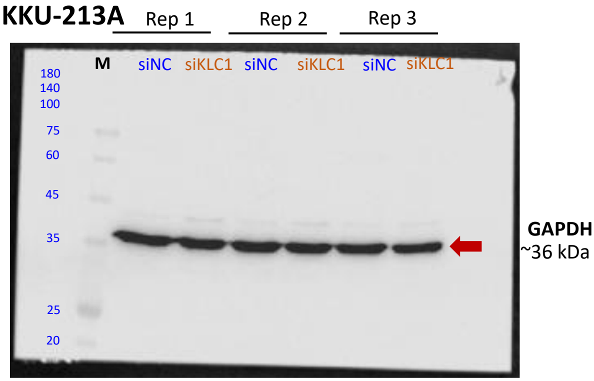

GAPDH protein detection: Total protein (30 µg/lane) from siKLC1-transfected KKU-213A cells was analyzed by Western blotting using mouse anti-GAPDH (1:10,000; Proteintech) and HRP-conjugated goat anti-mouse IgG (1:10,000; Abcam). Exposure time : 100 msec

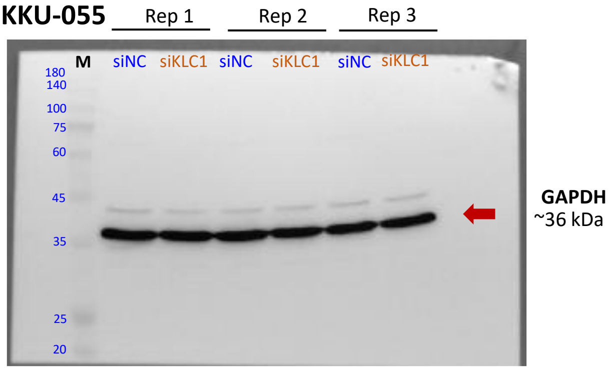

GAPDH protein detection: Total protein (30 µg/lane) from siKLC1-transfected KKU-055 cells was analyzed by Western blotting using mouse anti-GAPDH (1:10,000; Proteintech) and HRP-conjugated goat anti-mouse IgG (1:10,000; Abcam). Exposure time : 100 msec

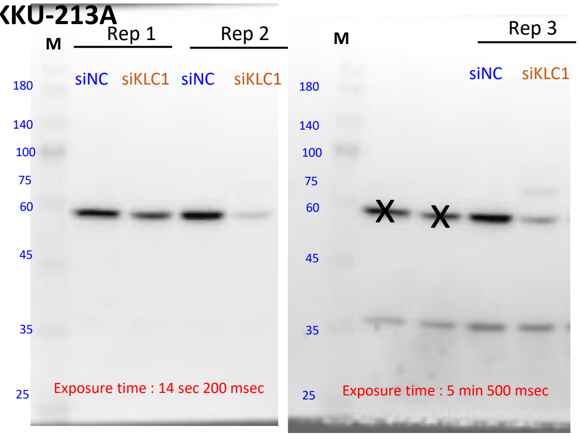

Vimentin protein detection: Total protein (30 µg/lane) from siKLC1-transfected KKU-213A cells was analyzed by Western blotting using rabbit anti-vimentin (1:5,000; Cell Signaling) and HRP-conjugated goat anti-rabbit IgG (1:10,000; Abcam).

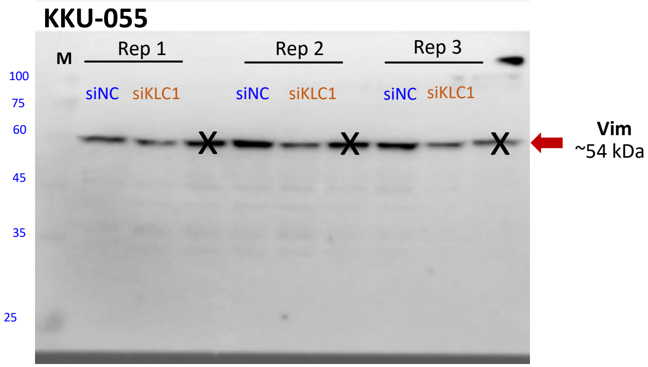

Vimentin protein detection: Total protein (30 µg/lane) from siKLC1-transfected KKU-055 cells was analyzed by Western blotting using rabbit anti-vimentin (1:5,000; Cell Signaling) and HRP-conjugated goat anti-rabbit IgG (1:10,000; Abcam). Exposure time : 28 sec 700 msec
